# Complete mitochondrial genome and phylogenetic analysis of the Weddell seal, *Leptonychotes weddellii*

**DOI:** 10.1080/23802359.2020.1820390

**Published:** 2020-09-15

**Authors:** Jong-U Kim, Jeong-Hoon Kim

**Affiliations:** Korea Polar Research Institute, Incheon, South Korea

**Keywords:** *Leptonychotes weddellii*, mitogenome, pinniped, Weddell seal

## Abstract

The Weddell seal, *Leptonychotes weddellii*, which belongs to the family Phocidae, is an abundant pinniped that inhabits the Antarctica. Here, we present the complete mitochondrial genome and phylogeny of *L. weddellii*. The total length of the mitogenome is 16,762 bp, consisting of 13 protein-coding genes (PCGs), 22 tRNA genes, and 2 rRNA genes. The base composition of the mitogenome is 34.26% (A), 25.51% (T), 27.09% (C), and 13.11% (G), and 40.22% for overall GC contents. The description of this mitogenome can provide information about variations at the intra-species level and aid phylogenetic studies in family Phocidae.

The Weddell seal (*Leptonychotes weddellii*) is one of the largest phocid seals inhabiting the coastal areas of Antarctica (Wheatley et al. 2006). This species shows the southernmost distribution and breeds on ice (Kooyman 1981). The breeding population is estimated as stable and is considered as Least Concern by the IUCN (Hückstädt 2015). The complete mitochondrial genome information of a species is important to understanding its mitogenomic evolution (Douglas and Gower 2010). Although the mitogenomic data of *L. weddellii* (AM181025) have been reported (Arnason et al. 2006), there is no specific information on the sampling location (described as only Antarctica). We tried to identify the intra-species mutation of mitogenome with more accurate geographical information. In this study, we sequenced and analyzed the mitogenome of *L. weddellii* and compared it with the results of the previous study and those of other species in the family Phocidae.

Here, we assembled the complete mitogenome using Illumina data and revealed the phylogeny of *L. weddellii* and its relationship with relative species. The mitogenome sequence was deposited in GenBank as MT755639. The carcass of *L. weddellii* was collected from Terra Nova Bay (74°38′15.69″S, 164°13′43.46″E), Ross Sea, Antarctica, on November 18, 2017. The specimen (proof number: WS01) was stored at the Korea Polar Research Institute (KOPRI), Incheon, South Korea. Total genomic DNA was isolated from muscle tissue using the DNeasy Blood and Tissue kit (Qiagen, Hilden, Germany), and then next-generation sequencing (NGS) was performed. High quality reads (>Q20) of 0.89 Gb were obtained from paired-end (PE) raw reads and were *de novo* assembled using the CLC genome assembler (v. 4.21, CLC Inc., Aarhus, Denmark) with a reads distance 400–600 bp, minimum length fraction for 0.5, and similarity for 0.8. The mitogenomes from the initially assembled contigs were processed to generate a single draft sequence (Lee et al. 2018). Overall sequences were annotated using GeSeq (https://chlorobox.mpimp-golm.mpg.de/geseq-app.html) and Artemis annotation tool (Rutherford et al. 2000) with NCBI BLASTN search (https://blast.ncbi.nlm.nih.gov). In addition, the mitogenome sequence variation was compared at the intra-species level between a reference mitogenome (AM181025) and a newly assembled mitogenome (MT755639). Two mitogenome sequences were aligned by MAFFT (Katoh and Standley 2013) and analyzed using single nucleotide polymorphism (SNP), and insertions and deletions (InDel) were detected. The results revealed 12 SNPs and 9 InDels between the two *L. weddellii* mitogenomes. Phylogenetic analysis was performed using the concatenated 13 PCGs set of the *L. weddellii* mitogenome with the inclusion of 16 published mitogenomes from the family Phocidae. A phylogenetic tree was constructed by the maximum likelihood (ML) method with 1000 bootstrap replicates via MEGA 7.0 program (Kumar et al. 2016) using the Tamura-Nei model (Figure 1).

**Figure 1.**
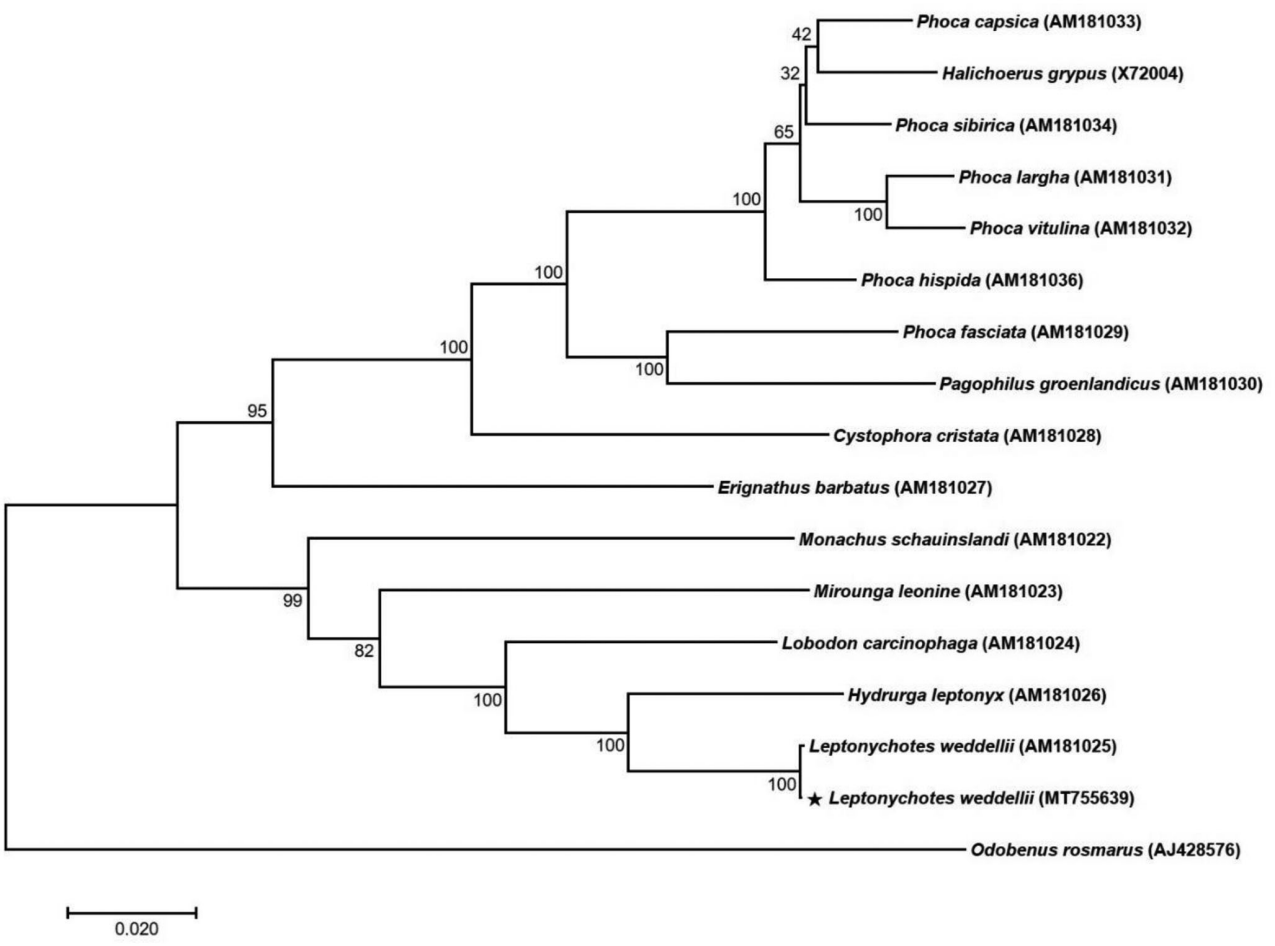
A phylogenetic tree of 14 species of the family Phocidae. Thirteen PCGs in the mitochondrial genome were aligned and used to generate the maximum likelihood phylogenetic tree. The numbers in the nodes indicate bootstrap support values (>50%) from 1,000 replicates.

The complete mitochondrial genome sequence of *L. weddellii* was 16,762 bp in length. A total of 37 genes were predicted in the genome, consisting of 13 protein-coding genes (PCGs), 22 tRNA genes, and 2 rRNA genes. The nucleotide composition of *L. weddellii* is as follows: 34.26% (A), 25.51% (T), 27.09% (C), and 13.11% (G), and 40.22% for overall GC contents. As expected, both *L.weddellii* genomes group together with 100% bootstrap in the ML tree.

## Data Availability

The data that support the finding of this study are openly available in NCBI at (https://www.ncbi.nlm.nih.gov), reference number [MT755639].
